# Supplementary material for: Cytoplasmic flow is a cell size sensor that scales anaphase
Source: Nat Cell Biol. 2025 Jan 31;27(2):273–82. doi: 10.1038/s41556-024-01605-6 (PMC11821524; doi:10.1038/s41556-024-01605-6)
Supplement: Supplementary file 1 — Supplementary Fig. 1, Discussion, Table 1 and References. [file 41556_2024_1605_MOESM1_ESM.pdf]

nature cell biology

Supplementary information

https://doi.org/10.1038/s41556-024-01605-6

Cytoplasmic flow is a cell size sensor that scales anaphase

In the format provided by the authors and unedited

1 Force balance on aster

In this part, we explore the separation dynamics of two interacting asters that are not confined. We consider two cases: the force on the asters is applied i) in the bulk or ii) at their interface. Throughout, we assume rotational invariance with respect to the axis connecting the two centers and apply a meanfield approach. The distance between the aster centers is $2d_a$, Sup. Fig. 1. We will assume a constant velocity v_g at which the aster radius r_a grows $\dot{r}_a = v_g$. First, we establish a relation between the force amplitude f_c acting on one aster and d_a for the two cases. Then, we determine an effective friction coefficient for an aster and finally analyze the dynamics.

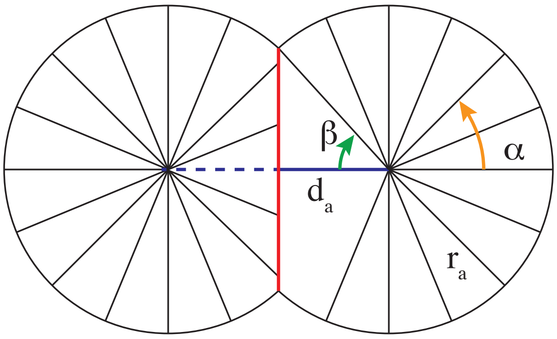

Supplementary Figure 1: Schematic representation of two interacting asters. Each aster is described as a circle of radius r_a with segments cut out as indicated in the scheme. The distance between the two aster centers is d_a. Microtubules are assumed to be radially oriented on average. The angle of the microtubules with the axis through the two aster centers is denoted by α and the opening angle of an aster is β.

1.1 Bulk forces

Before computing the relation between the force and the distance d_a, let us first study a possible origin of such a bulk force. When molecular motors transport a cargo, for example, a mitochondrion, along a microtubule, they exert the same, but oppositely directed force on the microtubule as on the cargo. Explicitly, force balance imposes

$$\mathbf{f}_{\mathrm{mito}} = -\mathbf{f}_{\mathrm{mt}}, \tag{1}$$

where $\mathbf{f}_{\mathrm{mito}}$ is the force on a mitochondrion and $\mathbf{f}_{\mathrm{mt}}$ that on a microtubule. In the low Reynolds number regime that we consider, these forces are related to the corresponding velocities by $\mathbf{f}_{\mathrm{mito}} = \xi_{\mathrm{mito}}\mathbf{v}_{\mathrm{mito}}$ and $\mathbf{f}_{\mathrm{mt}} = \xi_{\mathrm{mt}} \cdot \mathbf{v}_{\mathrm{mt}}$, respectively. The friction coefficient ξ_{mito} of a spherical mitochondrion with radius a_{mito} is $\xi_{\mathrm{mito}} = 6\pi\eta a_{\mathrm{mito}}$, where η is the viscosity of the surrounding fluid. Treating the microtubule as a slender rod, the friction coefficient is different when the microtubule is moved along its long axis or perpendicularly to it. Explicitly,

$$\mathbf{f}_{\mathrm{mt}} = \xi_{\mathrm{mt},\parallel}\mathbf{nn} \cdot \mathbf{v}_{\mathrm{mt}} + \xi_{\mathrm{mt},\perp}\left(\mathbf{1} - \mathbf{nn}\right) \cdot \mathbf{v}_{\mathrm{mt}} \tag{2}$$

with

$$\xi_{\mathrm{mt},\parallel} = 2\pi\eta L \frac{1}{\ln\left(L/2a_{\mathrm{mt}}\right) + \gamma_{\parallel}} \tag{3}$$

$$\xi_{\mathrm{mt},\perp} = 4\pi\eta L \frac{1}{\ln\left(L/2a_{\mathrm{mt}}\right) + \gamma_{\perp}}, \tag{4}$$

where L is the microtubule length, a_{mt} its radius, and $\gamma_{\parallel}$ and $\gamma_{\perp}$ are two constants [34]. Computations give a value of $\gamma_{\parallel}$ of the order of 0.1 and of $\gamma_{\perp}$ of the order 1, though precise values depend on details of the approach taken.

From these expressions, we can estimate the average distance between two mitochondria such that the velocity of the mitochondrion equals that of the microtubule. Indeed, if $L =$ 10µm and considering $a_{\text{mito}} \approx$ 0.5 µm and $a_{\text{mt}} \approx 12$ nm, we get $\xi_{\text{mt},\parallel} \approx \xi_{\text{mito}}$. Note that the force exerted by a molecular motor is directed along the microtubule's long axis. For a cytosolic viscosity of $\eta = 40\,\text{Pa s}$ [20], we get $\xi_{\text{mt},\parallel} \approx \xi_{\text{mito}} \approx 400\text{pN}/(\mu\text{m/s})$.

We now proceed to compute the total force acting on the center of an aster. Due to symmetry, the total force acts along the axis through the two aster centers. We assume that each molecular motor exerts the same force onto a microtubule and that this force is equal but opposite to $\mathbf{f} \equiv \xi_{\text{mito}}\mathbf{v}_{\text{mito}}$. The density of mitochondria is ρ_{mito}. As stated above, we assume an axisymmetric configuration and obtain for the force amplitude

$$f_c = 2\pi \int_0^{\pi-\beta} d\alpha \int_0^{r_a} dr\, r^2 \sin(\alpha)\cos(\alpha)\rho_{\text{mito}} f + 2\pi \int_{\pi-\beta}^{\pi} d\alpha \int_0^{-r_a \frac{\cos(\beta)}{\cos(\alpha)}} dr\, r^2 \sin(\alpha)\cos(\alpha)\rho_{\text{mito}} f \tag{5}$$

Since $r_a \cos(\beta) = d_a$, the first term can be rewritten as

$$2\pi f \int_{-d_a/r_a}^{1} \cos(\alpha)\, d\cos(\alpha) \int_0^{r_a} dr\, r^2 \rho_{\text{mito}}, \tag{6}$$

such that we obtain $\frac{\pi}{3}\left(r_a^2 - d_a^2\right) r_a \rho_{\text{mito}} f$ for $\rho_{\text{mito}} = const.$ For the second term, we get under the same assumption

$$-\frac{2\pi}{3} r_a^3 \rho_{\text{mito}} f \cos^3(\beta) \int_{\pi-\beta}^{\pi} d\alpha \frac{\sin(\alpha)}{\cos^2(\alpha)} = -\frac{2\pi}{3} r_a^3 \rho_{\text{mito}} f \cos^2(\beta)\left(1 - \cos(\beta)\right) \tag{7}$$

$$= -\frac{2\pi}{3} d_a^2 \left(r_a - d_a\right) \rho_{\text{mito}} f. \tag{8}$$

Consequently, the amplitude of the total force acting on the aster center is

$$f_c = \frac{\pi}{3}\left(1 - 3x^2 + 2x^3\right) r_a^3 \rho_{\text{mito}} f, \tag{9}$$

where $x \equiv d_a/r_a$ and $0 \le x \le 1$. For $x > 1$, we have $f_c = 0$. Note that our calculation does not account for hydrodynamic interactions between microtubules and/or dyneins.

1.2 Interfacial forces

Forces acting at the interface between the two asters (red line in Sup. Fig. 1) could result, for example, from steric interactions between microtubules or from molecular motors acting in the midzone. We assume that these forces are directed parallel to the axis through the two aster centers. The surface area of the interface is $\pi r_a^2 \sin^2(\beta)$. Assuming that the corresponding surface force density is uniform and equal to f in amplitude, we get

$$f_c = \pi r_a^2 \left(1 - x^2\right) f, \tag{10}$$

where again $x \equiv d_a/r_a$. The same, but opposite force acts on the other aster.

1.3 Dynamic equations

The dynamic equation for the half-distance d_a between the aster centers is generally given by

$$\xi_{\text{eff}} \dot{d}_a = f_c, \tag{11}$$

where f_c is the amplitude of the force acting on the aster center given either by Eq. (9) or Eq. (10). In the following, we determine the effective friction coefficient ξ_{eff}. As the force amplitude, also the effective friction coefficient is a function of r_a and d_a. We recall that the former is given by $r_a = v_g t$, such that the dynamic equation (11) is non-autonomous.

Integrating over the whole aster, we get

$$\xi_{\text{eff}} = \xi_0 2\pi \int_0^{\pi-\beta} d\alpha \int_0^{r_a} dr\, r^2 \sin(\alpha)\left(\cos(\alpha) + 2\sin(\alpha)\right)\rho_{\text{tub}} +$$
$$\xi_0 2\pi \int_{\pi-\beta}^{\pi} d\alpha \int_0^{-r_a \frac{\cos(\beta)}{\cos(\alpha)}} dr\, r^2 \sin(\alpha)\left(\cos(\alpha) + 2\sin(\alpha)\right)\rho_{\text{tub}}, \tag{12}$$

where ξ_0 is the friction coefficient of a small piece of a microtubule. In terms of the opening angle β, we obtain

$$\xi_{\rm eff} = \frac{\pi}{3}\left[1 + 2\pi - 2\beta + \cos^2(\beta) + \cos^3(\beta)\left(\ln\left(\frac{2}{1-\sin(\beta)} - 1\right) - 2\right)\right] r_a^3 \rho_{\rm tub}\xi_0. \tag{13}$$

For an opening angle $0 \le \beta \le \pi/4$, this expression is only weakly dependent on β (less than 4% variation). Note that, in the case of bulk forces, both, the total force on the aster center f_c, Eq. (9), and the friction coefficient $\xi_{\rm eff}$, Eq. (13), scale as r_a^3, such that aster growth affects both quantities equally to leading order. The effects on the two quantities thus compensate each other.

Neglecting the dependence on the opening angle and for the bulk force Eq. (9), one obtains

$$\dot{d}_a = v_0(1 - 3d_a^2/r_a^2 + 2d_a^3/r_a^3) \tag{14}$$

$$\dot{r}_a = v_g, \tag{15}$$

where v_0 is the separation velocity of the asters, when their centers colocalize. Asters separate at constant velocity if $\dot{d}_a = \dot{r}_a = const.$ This situation is stable, because $\dot{d}$ monotonically decreases with d_a. Thus, if d_a grows too fast, it will slow down and *vice versa*. This analysis can be generalized to the case that we retain the form (13) of the effective friction coefficient.

Under the same assumption in the case of interfacial forces, we get

$$\dot{d}_a = v_0\left(1 - d_a^2/r_a^2\right)\frac{r_0}{r_a} \tag{16}$$

$$\dot{r}_a = v_g, \tag{17}$$

where r_0 is some length. There is no solution such that $\dot{d}_a = \dot{r}_a = const.$

2 Confinement effect

To investigate the influence of membrane-induced confinement on chromosome dynamics, we employ numerical computations. We describe the asters as droplets of an additional fluid phase immersed in a solvent accounting for the cytosol. They experience pulling by a bulk force representing the action of dynein motors on the aster. We assume the chromosomes to be advected with the fluid flow and do not include them explicitly in the description.

2.1 Dynamic equations

We consider an incompressible fluid with three components: the solvent (cytosol) of mass density ρ_s and velocity $\mathbf{v}_s$; and two phase-fields (asters) of concentration $\phi_{1,2}$ and velocity $\mathbf{v}_{\phi_{1,2}}$. The total mass density is $\rho = \rho_s + \phi_1 + \phi_2$, which we assume to be constant, and the center of mass velocity $\mathbf{v} = (\rho_s\mathbf{v}_s + \phi_1\mathbf{v}_{\phi_1} + \phi_2\mathbf{v}_{\phi_2})/\rho$. The total fluid momentum is denoted $g_\alpha = \rho v_\alpha$.

The continuity equations for the three components are

$$\partial_t\rho_s + \partial_\alpha J_\alpha^s = 0 \tag{18}$$

$$\partial_t\phi_{1,2} + \partial_\alpha J_\alpha^{\phi_{1,2}} = 0 \tag{19}$$

$$\partial_t g_\alpha - \partial_\beta\sigma_{\alpha\beta} = f_\alpha^{ext} \tag{20}$$

with J_α^s, $J_\alpha^{\phi_1}$, $J_\alpha^{\phi_2}$ being the mass density current of respectively the solvent, aster 1 and aster 2. The mass density current can be split in two parts, the convective and the diffusive part j_α,

$$J_\alpha^s = \rho_s v_\alpha - j_\alpha^{\phi_1} - j_\alpha^{\phi_2} \tag{21}$$

$$J_\alpha^{\phi_{1,2}} = \phi_{1,2}v_\alpha + j_\alpha^{\phi_{1,2}} \tag{22}$$

$$J_\alpha = J_\alpha^s + J_\alpha^{\phi_1} + J_\alpha^{\phi_2} = \rho\mathbf{v}. \tag{23}$$

The free energy associated with the phase fields is given by

$$F = \int d^3\mathbf{r}\left\{\frac{a}{4\phi_{cr}^4}\phi_1^2(\phi_1 - \phi_0)^2 + \frac{k}{2}\left|\nabla\phi_1\right|^2 + \frac{a}{4\phi_{cr}^4}\phi_2^2(\phi_2 - \phi_0)^2 + \frac{k}{2}\left|\nabla\phi_2\right|^2 + \frac{\lambda}{2}\phi_1^2\phi_2^2\right\} \tag{24}$$

with positive constants a and k. The corresponding terms in the free energy, respectively, fix the mean density in the aster phase and penalize gradients. The latter is equal to the asters surface tension. Finally, λ represents

the affinity or repulsion between the two phase-fields. Since in the mid-zone asters repel each other in fish embryos, λ is chosen positive. The chemical potentials for the phase fields are defined as

$$\mu_{1,2} = \frac{\delta F}{\delta \phi_{1,2}} = \frac{\partial f}{\partial \phi_{1,2}} - \partial_\alpha \frac{\partial f}{\partial \partial_\alpha \phi_{1,2}} \tag{25}$$

The hydrostatic stress is given by

$$\sigma_{\alpha\beta} = (f - \phi_1 \mu_1 - \phi_2 \mu_2)\delta_{\alpha\beta} - \frac{\partial f}{\partial \partial_\beta \phi_1} \partial_\alpha \phi_1 - \frac{\partial f}{\partial \partial_\beta \phi_2} \partial_\alpha \phi_2. \tag{26}$$

To ensure incompressibility in our computational scheme, we add an isotropic pressure term $p = (c^2/3)\rho$, which is kept large compare to the other terms in the momentum conservation.

With these definitions, the dynamic equations read

$$\rho(\partial_t + v_\beta \partial_\beta) v_\alpha = -\partial_\beta (c^2 \rho \delta_{\alpha\beta}) + \partial_\beta (\sigma_{\alpha\beta} + \eta(\partial_\beta v_\alpha + \partial_\alpha v_\beta)) + f_m \left(\frac{\phi_1}{\int \mathrm{d}^3\mathbf{r} \phi_1} - \frac{\phi_2}{\int \mathrm{d}^3\mathbf{r} \phi_2} \right) \delta_{\alpha x} \tag{27}$$

$$\partial_t \phi_{1,2} + \partial_\alpha (v_\alpha \phi_{1,2}) = M \nabla^2 \mu_{1,2} + k_p \left| \nabla \phi_{1,2} \right|^2 (\phi_0 - \phi_{2,1}) \tag{28}$$

Here, η is viscosity and f_m is the amplitude of the force exerted on the phase fields. This force is directed along the x-axis, which coincides with the axis through the aster centers.

In the continuity equations for the phase fields, M is the mobility and k_p the growth rate of the asters. The factor $|\nabla \phi_{1,2}|^2$ in the corresponding term confines growth to the boundary of the phase-field boundary, whereas the second one $(\phi_0 - \phi_{2,1})$ prevents a growing phase field to invade the other aster.

As stated above, the position $\mathbf{x}_{1,2}$ of the chromosomes evolves through advection with the fluid. Explicitly, we can write

$$\frac{d}{dt} \mathbf{x}_{1,2} = \mathbf{v}(\mathbf{x}_{1,2}) \tag{29}$$

We use no-slip boundary conditions for the velocity at the cell boundary and the growth rate of the aster ceases where it touches the boundary to not grow outside: $k_p = \tilde{k}_p(\psi - 1.0)$. Here ψ is a static phase-field such that $\psi = 2.0$ ($\psi = 0.0$) inside (outside) the cell.

2.2 Numerical methods

The total mass density and velocity of the fluid (ρ, v) are computed using the lattice Boltzmann method with the D2Q9 lattice and the single-relaxation-time (BGK) collision operator. The force is projected on lattice velocities using the second order Guo force scheme. The no-slip boundary condition is implemented using the half-way Bounce-Back method. Once the velocity is computed, the temporal evolution of the phase field is computed using the up-wind scheme for advection and a second order central difference for diffusion. The temporal integration is made using an explicit Euler forward scheme. The dynamic of the chromosomes are computed using the Euler forward method.

The Reynolds number is kept low (Re < 1) in order to not have inertia effects.

Mapping of parameters: The discretization is such that the physical $\Delta x = 1\mu$m is equal to 1 ($\delta x = 1$) in lattice Boltzmann units. The maximal velocity in the system is the velocity of the chromosomes $|\mathbf{v}^{max}| = 0.1\mu$m/s. In order to ensure incompressibility and stability of the algorithm the maximal speed in lattice units have to be small $|\mathbf{v}_{LB}^{max}| = |\mathbf{v}^{max}| \Delta t / \Delta x \sim 10^{-4}$, fixing $\Delta t \sim 10^{-3}$s. The time-step in lattice unit is $\delta t = 1$.

The size of the cell, its shape, the initial size of the aster, and the distance between the chromosomes at onset of anaphase B are set corresponding to their biological values. The growth rate of the aster $\tilde{k}_p$ and the parameter of the force f_m are fitted to our experimental observations of the aster growth and the velocity of chromosomes in absence confinement.

Simulation parameters: $\rho = 1.0$, $a = 1 \cdot 10^{-7}$, $k = 2 \cdot 10^{-7}$, $\lambda = 1 \cdot 10^{-8}$, $M = 1 \cdot 10^6$, $f_m = 1.8 \cdot 10^{-5}$, $\tilde{k}_p = 0.2$, the aster radius $R_{\mathrm{aster}}^{\mathrm{ini}} = 20.5$, aspect ratio between the long and short axis 1.4, initial distance between asters $R_{\mathrm{aster}}/2$, initial distance between chromosomes $d_{\mathrm{ch}} = 18$. The relaxation time is computed as $\tau = \frac{6R_{\mathrm{aster}}^{\mathrm{ini}} |\mathbf{v}^{max}|}{\tilde{\mathrm{Re}}} + \frac{1}{2}$ with the estimation of the Reynolds number $\tilde{\mathrm{Re}} = 1$. All parameters used are listed in Table 1 of the Supplementary Material.
