# Supplementary material for: Cytoplasmic flow is a cell size sensor that scales anaphase
Source: Nat Cell Biol. 2025 Jan 31;27(2):273–82. doi: 10.1038/s41556-024-01605-6 (PMC11821524; doi:10.1038/s41556-024-01605-6)
Supplement: Supplementary file 2 — Reporting Summary [file 41556_2024_1605_MOESM2_ESM.pdf]

nature portfolio

Corresponding author(s): Olga Afonso
Marcos Gonzalez-Gaitan

Last updated by author(s): Oct 11, 2024

# Reporting Summary

Nature Portfolio wishes to improve the reproducibility of the work that we publish. This form provides structure for consistency and transparency in reporting. For further information on Nature Portfolio policies, see our Editorial Policies and the Editorial Policy Checklist.

## Statistics

For all statistical analyses, confirm that the following items are present in the figure legend, table legend, main text, or Methods section.

| n/a | Confirmed | |
|---|---|---|
| ☐ | ☒ | The exact sample size ($n$) for each experimental group/condition, given as a discrete number and unit of measurement |
| ☒ | ☐ | A statement on whether measurements were taken from distinct samples or whether the same sample was measured repeatedly |
| ☐ | ☒ | The statistical test(s) used AND whether they are one- or two-sided<br>*Only common tests should be described solely by name; describe more complex techniques in the Methods section.* |
| ☒ | ☐ | A description of all covariates tested |
| ☐ | ☒ | A description of any assumptions or corrections, such as tests of normality and adjustment for multiple comparisons |
| ☐ | ☒ | A full description of the statistical parameters including central tendency (e.g. means) or other basic estimates (e.g. regression coefficient) AND variation (e.g. standard deviation) or associated estimates of uncertainty (e.g. confidence intervals) |
| ☐ | ☒ | For null hypothesis testing, the test statistic (e.g. $F$, $t$, $r$) with confidence intervals, effect sizes, degrees of freedom and $P$ value noted<br>*Give P values as exact values whenever suitable.* |
| ☒ | ☐ | For Bayesian analysis, information on the choice of priors and Markov chain Monte Carlo settings |
| ☒ | ☐ | For hierarchical and complex designs, identification of the appropriate level for tests and full reporting of outcomes |
| ☒ | ☐ | Estimates of effect sizes (e.g. Cohen's $d$, Pearson's $r$), indicating how they were calculated |

*Our web collection on statistics for biologists contains articles on many of the points above.*

## Software and code

Policy information about availability of computer code

| | |
|---|---|
| Data collection | Software built in the microscope for image acquisition: Slidebook 6 (3i) and Zeiss Zen. Numerical simulations were performed with custom written code on Julia. |
| Data analysis | FiJi (2.9), Microsoft Excel for Mac 2023, Prism 10 and MATLAB 2021b (The MathWorks) software were used for analysis. Figures were generated using Affinity Photo v1 and Affinity Publisher v1.<br>Numerical simulations were performed in Julia v1.10.5 with CUDA.jl and Makie.jl packages (versions detailed on GitHub - https://github.com/Lu-Dumoulin/SM_OA_MGG.git).<br>MSD analyzer (https://ch.mathworks.com/matlabcentral/fileexchange/40692-mean-square-displacement-analysis-of-particles-trajectories) was used to compute MSD analysis. Shaddederrorbar package (https://ch.mathworks.com/matlabcentral/fileexchange/26311-raacampbell-shadederrorbar) was used to generate shade of standard error on figures.<br>Trackmate plugin on Fiji was used to analyse EB3 comets and tubulin speckles (https://imagej.net/plugins/trackmate/)<br>PIVlab 2.55, Matlab package, was used to analyse cytoplasmic flows. |

For manuscripts utilizing custom algorithms or software that are central to the research but not yet described in published literature, software must be made available to editors and reviewers. We strongly encourage code deposition in a community repository (e.g. GitHub). See the Nature Portfolio guidelines for submitting code & software for further information.

## Data

Policy information about availability of data

All manuscripts must include a data availability statement. This statement should provide the following information, where applicable:

- Accession codes, unique identifiers, or web links for publicly available datasets
- A description of any restrictions on data availability
- For clinical datasets or third party data, please ensure that the statement adheres to our policy

Source data are provided with this study. All other data supporting the findings of this study are available from the corresponding authors upon reasonable request.

## Research involving human participants, their data, or biological material

Policy information about studies with human participants or human data. See also policy information about sex, gender (identity/presentation), and sexual orientation and race, ethnicity and racism.

| | |
|---|---|
| Reporting on sex and gender | n/a |
| Reporting on race, ethnicity, or other socially relevant groupings | n/a |
| Population characteristics | n/a |
| Recruitment | n/a |
| Ethics oversight | n/a |

Note that full information on the approval of the study protocol must also be provided in the manuscript.

# Field-specific reporting

Please select the one below that is the best fit for your research. If you are not sure, read the appropriate sections before making your selection.

☒ Life sciences ☐ Behavioural & social sciences ☐ Ecological, evolutionary & environmental sciences

For a reference copy of the document with all sections, see nature.com/documents/nr-reporting-summary-flat.pdf

# Life sciences study design

All studies must disclose on these points even when the disclosure is negative.

| | |
|---|---|
| Sample size | Sample size were not pre-calculated to perform our experiments. Sample sizes were based on comparison between control and test conditions where the statistical significances could be efficiently measured. |
| Data exclusions | As mentioned in the methods section of the manuscript, embryos that showed errors during mitosis were excluded from the analysis. In experimental conditions where errors in mitosis are a consequence of the experiment itself (drug conditions such as SbTub3P, dynein, Cdk1 and actin inhibitions), this exclusion didn't apply. |
| Replication | Adult zebrafish of the relevant genetic background but from different batches and generations where chosen to mate. Embryos were randomly picked from each spawning. Thus each embryo was considered an independent biological experiment. For each condition at least two independent biological experiments were performed. |
| Randomization | Zebrafish with the same AB genetic background were used, but from different batch and generations and experiments were carried out on randomly chosen zebrafish embryos of the appropriate genetic background and developmental stage. Our study does not explore the impact of different treatments on subjects, nor did it require sampling individuals that belong to different groups from large populations. As such randomization is not strictly relevant to our analysis. |
| Blinding | No blinded studies were performed. Blinding is not relevant for this study as most conditions are analysis of wild type embryos. For experiments with drugs, the technical experiment and the analysis were performed by the same investigator. |

# Reporting for specific materials, systems and methods

We require information from authors about some types of materials, experimental systems and methods used in many studies. Here, indicate whether each material, system or method listed is relevant to your study. If you are not sure if a list item applies to your research, read the appropriate section before selecting a response.

## Materials & experimental systems

| n/a | Involved in the study | |
|---|---|---|
| ☐ | ☒ | Antibodies |
| ☒ | ☐ | Eukaryotic cell lines |
| ☒ | ☐ | Palaeontology and archaeology |
| ☐ | ☒ | Animals and other organisms |
| ☒ | ☐ | Clinical data |
| ☒ | ☐ | Dual use research of concern |
| ☒ | ☐ | Plants |

## Methods

| n/a | Involved in the study | |
|---|---|---|
| ☒ | ☐ | ChIP-seq |
| ☒ | ☐ | Flow cytometry |
| ☒ | ☐ | MRI-based neuroimaging |

# Antibodies

Antibodies used

- rabbit anti-pH3-s10 D2C8, 1:200 (Cell Signalling, ref. 3377, lot7)
- Fab antibody against phosphorylation at S10 of Histone H3 conjugated with Alexa-488, Alexa-Cy3 or Alexa-Cy5 (1:10 from 2ug/ml stock). Kind gift from Hiroshi Kimura lab, Tokyo Tech, Japan.

Validation

- rabbit anti-pH3-s10 D2C8 - validated by the supplier

- Fab antibody - validated previously by Hayashi-Takanaka, Y., Yamagata, K., Nozaki, N. & Kimura, H. Visualizing histone modifications in living cells: spatiotemporal dynamics of H3 phosphorylation during interphase. J Cell Biol 187, 781-790 (2009).

# Animals and other research organisms

Policy information about studies involving animals; ARRIVE guidelines recommended for reporting animal research, and Sex and Gender in Research

Laboratory animals

Adult zebrafish between 3 months and 18 months old were used for mating and the offspring (zebrafish embryos up to 3hpf) was used in this study. Strains used are: Tg(h2afva:h2afva-GFP), Tg(Ef1alpha:H2B-mCherry), Tg(Ef1alpha:MLS-GFP), Tg(actb1:Utr-GFP), Tg(actb1:Utr-mCherry), Tg(bactin2:HsENSCONSIN17-282-3xEGFP), Tg(actb2:EGFP-Has.DCX) and Tg(-5bactin2:cetn2-GFP)

Wild animals

No wild animals were used in the study.

Reporting on sex

This study was performed in embryos up to 3hpf where sex is not yet determined. Sex of the animals is not relevant for this study.

Field-collected samples

No field collected samples were used in the study.

Ethics oversight

Housing and ethic are and monitored regularly by the Swiss veterinary office (https://www.blv.admin.ch/blv/en/home/tiere/tierversuche.html)

Note that full information on the approval of the study protocol must also be provided in the manuscript.

# Plants

Seed stocks

n/a

Novel plant genotypes

n/a

Authentication

n/a
